# Circulating Tumor DNA Analysis on Metastatic Prostate Cancer with Disease Progression

**DOI:** 10.3390/cancers15153998

**Published:** 2023-08-07

**Authors:** Sungun Bang, Dongju Won, Saeam Shin, Kang Su Cho, Jae Won Park, Jongsoo Lee, Young Deuk Choi, Suwan Kang, Seung-Tae Lee, Jong Rak Choi, Hyunho Han

**Affiliations:** 1 Department of Urology, Severance Hospital, Urological Science Institute, Yonsei University College of Medicine, Seoul 03722, Republic of Korea; bbsungun@yuhs.ac (S.B.); js1129@yuhs.ac (J.L.); youngd74@yuhs.ac (Y.D.C.);; 2 Department of Urology, National Health Insurance Service Ilsan Hospital, Goyang 10444, Republic of Korea; epria@nhimc.or.kr; 3 Department of Laboratory Medicine, Severance Hospital, Yonsei University College of Medicine, Seoul 03722, Republic of Korea; saeam0304@yuhs.ac (S.S.); lee.st@yuhs.ac (S.-T.L.); cjr0606@yuhs.ac (J.R.C.); 4 Department of Urology, Prostate Cancer Center, Gangnam Severance Hospital, Urological Science Institute, Yonsei University College of Medicine, Seoul 03722, Republic of Korea; kscho99@yuhs.ac

**Keywords:** circulating tumor DNA, liquid biopsy, metastasis, prostate cancer

## Abstract

**Simple Summary:**

In our study, we found genetic mutations in the DNA of cancer cells obtained from the blood of many patients with metastatic prostate cancer. These changes were more common in patients whose cancer was progressing and aggressive in nature, often unresponsive to conventional treatment strategies. Additionally, these genetic mutation findings were more sensitive than the conventional method of checking the prostate-specific antigen (PSA) level in the blood for detecting prostate cancer progression. This means a simple blood test could help us track genetic mutations, detect aggressive cancer subtypes, determine the progression of the disease, and manage advanced prostate cancer more effectively.

**Abstract:**

The positivity rate of circulating tumor DNA (ctDNA) next-generation sequencing (NGS) varies among patients with metastatic prostate cancer (mPC), complicating its incorporation into regular practice. This retrospective study analyzed the ctDNA sequencing results of 100 mPC patients from May 2021 to March 2023 to identify the factors associated with positive ctDNA. Three custom gene panels were used for sequencing. Overall, 63% of the patients exhibited tier I/II somatic alterations, while 12% had pathogenic/likely pathogenic germline alterations. The key genes that were altered included *AR*, *TP53*, *RB1*, *PTEN*, and *APC*. Mutations in *BRCA1/2*, either germline or somatic, were observed in 21% of the patients. Among the metastatic castration-resistant prostate cancer (mCRPC) patients, the ctDNA-positive samples generally showed higher median prostate-specific antigen (PSA) levels and were more likely to be at the radiographic and clinical progressive disease stages, although they were not significantly associated with PSA progression. Our results suggest that ctDNA analysis could detect meaningful genetic changes in mPC patients, especially during disease progression.

## 1. Introduction

The multitude of treatment options makes drug selection and sequencing a clinical challenge in the management of metastatic castration-resistant prostate cancer (mCRPC) and metastatic hormone-sensitive prostate cancer (mHSPC) [1]. Androgen deprivation therapy (ADT) is primarily used to treat mHSPC [2], and docetaxel or androgen signaling inhibitors (ARSIs) are included in the standard of care for both mHSPC and mCRPC along with ADT [2]. In addition, new treatments for mCRPC have emerged recently, such as Poly (ADP-ribose) polymerase (PARP) inhibitors, immune checkpoint inhibitors, and prostate-specific membrane antigen (PSMA) radioligands targeted for certain genetic or radiological profiles [3,4,5,6].

Conventional diagnostic and monitoring tools for prostate cancer include serum PSA, CT, MRI, bone scans, and tissue biopsies. However, these methods may not detect all disease progressions, and the newly emerging systemic treatments require genomic biomarkers for treatment decision making [2,7,8]. Currently, most clinically available genomic tests are based on next-generation sequencing (NGS) and utilize either archived or freshly obtained tumor tissue. However, archived tissues have quality issues of degradation over time and may not be available. Fresh tissue is ideal for NGS genomic testing but is difficult to acquire in metastatic sites such as bone or deep lymph nodes. Additionally, tumor biopsies may not fully represent important tumor characteristics due to tumor heterogeneity and are not well-suited for serial monitoring.

Circulating tumor DNA (ctDNA) holds promise as a noninvasive method for the real-time assessment of a tumor’s genetic profile [9]. Recent clinical trials have associated ctDNA positivity at presentation with poor responses to systemic therapy [10,11]. Moreover, ctDNA quantity and dynamics can offer additional prognostic insights into the survival of patients with mPC [12]. These findings suggest that a blood-based liquid biopsy may substitute a tumor tissue biopsy for the management of mHSPC and mCRPC. However, the fact that ctDNA analyses may not reveal all the critical genetic alterations remains a concern, limiting its use in clinical practice compared to traditional tissue biopsies [13].

We recently initiated NGS-based testing for somatic and germline genetic alterations in plasma-cell-free DNA (cfDNA) and matched leukocyte genomic DNA (gDNA) from patients with advanced prostate cancer. As the samples were collected across different disease stages and during varying therapies, we hypothesized that reviewing the ctDNA data alongside the patient’s biochemical, radiographic, or clinical characteristics can be useful for the successful management of mPC. Here, we present real-world ctDNA data from 100 cases of mPC, focusing on “when” the ctDNA analysis provides useful genomic information.

## 2. Materials and Methods

### 2.1. Patient Population

This study was approved by the Severance Hospital Bioethics Committee (No. 4-2021-0276) for plasma sample collection and evaluation of biomarker function. Medical records were gathered from 100 patients who received treatment for mCRPC or mHSPC and underwent ctDNA NGS assays as part of their clinical care in the Yonsei University Health System (Seoul, Republic of Korea) between May 2021 and March 2023. Metastasis was identified by lesions detected in bone scans, computed tomography (CT), magnetic resonance imaging (MRI), or fluorodeoxyglucose (FDG) positron emission tomography–`CT (PET-CT). PSMA PET-CT was not used as a sole criterion to define metastasis. HSPC was defined as the absence of ADT use from the time of diagnosis to the sampling date or for the past two years, or it was defined by nonrising PSA levels and radiographically stable disease under ADT. Furthermore, CRPC was defined as a history of consecutive PSA increases or radiographic progression despite ADT. Patients with active malignancies other than prostate cancer; nonmetastatic prostate cancer; or insufficient data to determine PSA, radiographic, or clinical response to therapy were excluded from further analysis. Patients enrolled in multiarm clinical trials at the time of sampling were also excluded.

This study was conducted in accordance with the Declaration of Helsinki and the Good Clinical Practice Guidelines of the International Conference of Harmonization. Written informed consent was obtained from the patients included in this study for the publication of their case details.

### 2.2. Clinical Data Collection

We collected prostate-cancer-relevant clinical data, including Gleason score and histological subtype, which were obtained from prostate needle biopsy, prostatectomy, metastatic biopsy, and palliative transurethral resection of the prostate (TURP). Serum PSA levels, CT, MRI, bone scans, PET-CT data, and other relevant medical records at the time of sampling were retrieved. The history of systemic treatment for prostate cancer included ADT, abiraterone, enzalutamide, docetaxel, cabazitaxel, radiation therapy, and participation in clinical trials. Progressive disease was defined biochemically and radiographically according to the Prostate Cancer Clinical Trials Working Group 3 (PCWG3). Clinical progression was defined as worsening cancer-related pain or clinical course deterioration, such as gross hematuria, urinary retention, spinal compression symptoms, or cancer-related deaths.

### 2.3. ctDNA Somatic and Germline Variant Analysis

In this study, we used three sets of custom ctDNA analysis panels: the PAN100 panel targeting 101 genes detected in malignant tumors, the TMB500 panel targeting 531 genes detected in malignant tumors [14], and the PC panel targeting 54 genes detected in prostate cancer (Supplementary Table S3). For sampling, we collected a total of 18 mL or 9 mL of whole blood using a Dxtube (Dxome, Seongnam, Gyeonggi-do, Republic of Korea) containing preservation solutions. The blood sample was centrifuged for 15 min at 1900× *g*, and the supernatant was centrifuged again for 10 min at 1900× *g*. CfDNA was extracted from plasma using the Magnetic Circulating DNA Maxi Reagent (Dxome). gDNA was extracted using the QIAamp DNA Mini Kit (QIAGEN, Hilden, Germany) from buffy coat derived from matched whole blood. Size and quantitative measurements were performed using the D1000 ScreenTape system (Agilent, CA, USA). Library preparation was carried out using the Library Prep Reagent for Illumina (Dxome). Paired-end sequencing was performed using NovaSeq 6000 system (Illumina, CA, USA). The FASTQ files were aligned to the reference genome of GRCh37 (hg19) using Burrows–Wheeler alignment (BWA) tool [15]. Single-nucleotide variants (SNVs) and small indels were identified using the PiSeq algorithm (Dxome), which adopts the genome position of the sequencing reads and refines the accuracy of molecular barcoding to enable accurate determination of variants with low variant allele frequency, and were visually confirmed using Integrated Genomics Viewer. Copy number variations (CNVs) were detected by ExomeDepth [16] and CABANA algorithms [17]. Variants in both cfDNA and gDNA were determined as germline variants or variants of clonal hematopoiesis of indeterminate potential. Somatic variants were classified into four tiers based on the standards and guidelines of the Association for Molecular Pathology (AMP), American Society of Clinical Oncology (ASCO), and College of American Pathologists (CAP) [18]. Germline variants were classified as pathogenic, likely pathogenic, or of unknown significance according to the guidelines of the American College of Medical Genetics and Genomics (ACMG) and the AMP [19,20].

### 2.4. Statistical Analysis

The primary objective of this study was to compare ctDNA positivity or negativity with disease progression status at the time of sampling. CtDNA positivity in this study was defined by the detection of tier I or II somatic variants, while ctDNA negativity referred to their absence. The patients were divided into ctDNA-positive and ctDNA-negative groups. The difference in PSA levels between the two groups was analyzed using the Mann–Whitney U test. Fisher’s exact test was used to examine whether the two groups showed statistically significant differences in PSA, radiographic, or clinical progression. Statistical significance was set at *p* < 0.05.

## 3. Results

### 3.1. Patient Characteristics

Table 1 lists the demographic data and characteristics of the patients (refer to Supplementary Table S1 for original data). We collected data from 77 patients with mCRPC and 23 patients with mHSPC for a ctDNA data analysis. Among the patients with mCRPC, 40 (51.9%) had asynchronous metastases, while all the patients with mHSPC (100.0%) demonstrated synchronous metastases. From the biopsy or prostatectomy samples, 92 (92.0%) patients had cancers of Gleason grade group 4 or 5. Within the mCRPC cohort, 32 (41.6%) received docetaxel or cabazitaxel chemotherapies, and 53 (68.8%) were treated with one or more ARSIs. Both chemotherapy and ARSIs were administered to 21 patients (27.2%). Two patients (2.5%) participated in clinical trials involving PARP inhibitors prior to ctDNA sampling, but their participation was terminated due to disease progression.

### 3.2. Somatic Mutation Profiles of ctDNA in Metastatic Prostate Cancer

**Figure 1:**
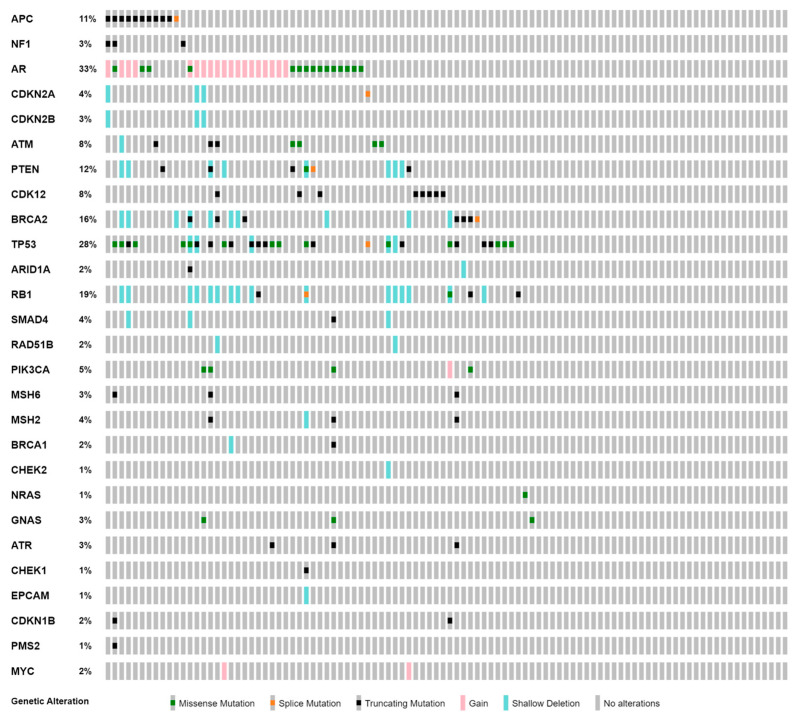
Somatic mutation profile of ctDNA in metastatic prostate cancer.

The average depth per sample was over 30,000×. Among the patients with mPC, sixty-nine (69.0%) had AMP/ASCO/CAP tier I (with strong clinical significance) or tier II (with potential clinical significance) somatic variants. Since we used three gene panels in this study, our analysis was limited to the 35 genes shared by all three panels (Supplementary Table S3). Frequently altered genes included *AR* (33.0%), *TP53* (28.0%), *RB1* (19.0%), *BRCA2* (16.0%), *PTEN* (12.0%), and *APC* (11.0%) (Figure 1). As expected, *AR* alterations were exclusively observed in mCRPCs (4.4% vs. 41.6%, *p* = 0.007, Fisher’s exact test). The frequencies of *TP53*, *RB1*, *BRCA2*, *PTEN,* or *APC* alterations were not significantly different between mHSPCs and mCRPCs (Supplementary Table S2).

### 3.3. BRCA1 and BRCA2 Mutation Frequencies in Metastatic Prostate Cancer

Fourteen (14.0%) pathogenic or likely pathogenic germline alterations were identified in the mPC cohort. The most frequently altered gene was *BRCA2* (7%), and *BRCA1*, *BRIP1*, *CHEK2*, *FANCA*, *ATM*, *MSH2,* and *MSH6* were each found in one patient. Importantly, following the addition of somatic and germline profiles, 23 (23%) patients showed *BRCA1* or *BRCA2* genetic alterations (tier I/II in somatic variants and pathogenic/likely pathogenic in germline variants) (Table 2, Supplementary Table S4).

#### BRCA1/2-Altered Metastatic Prostate Cancer’s Clinical and Pathological Characteristics

We compared the clinical characteristics of the 23 cases of *BRCA1/2* oncogenic alterations (*BRCA1/2*_mut_) with the remaining 77 wild-type (*BRCA1/2*_WT_) cases. There were no significant differences in patient age or TNM stage distribution between the *BRCA1/2*_mut_ and *BRCA1/2*_WT_ groups (Table 3). Interestingly, the mean PSA at the time of diagnosis in the *BRCA1/2*_mut_ group tended to be lower than that in the *BRCA1/2*_WT_ group (median PSA of 55.8 ng/mL vs. 117.1 ng/mL, *p* = 0.123, Table 3). Although not statistically significant, Gleason grade group (GGG) 5 tumors were more frequent in the *BRCA1/2*_mut_ group compared to the *BRCA1/2*_WT_ group (69.6% vs. 47.4%, *p* = 0.094, Table 3).

We separated the cohort into the synchronous metastasis (M1 at presentation, n = 64) and asynchronous disease (M0 at presentation, n = 35) subgroups. One patient did not have the relevant medical records for clinical staging at presentation. In the synchronous metastasis subgroup, the median PSA at presentation was significantly lower in the *BRCA1/2*_mut_ group than in the *BRCA1/2*_WT_ group (61.8 ng/mL vs. 224.4 ng/mL, *p* = 0.034, Mann–Whitney test, Table 4). In contrast, in the asynchronous metastasis subgroup, serum PSA tended to be higher in the *BRCA1/2*_mut_ group than in the *BRCA1/2*_WT_ group (50.9 ng/mL vs. 20.0 ng/mL, *p* = 0.224, Mann–Whitney test, Table 5).

### 3.4. Clinical and Pathological Characteristics of Aggressive-Variant Prostate Cancer (AVPC)

Aggressive-variant prostate cancer (AVPC) is clinically defined by an advanced T/N stage at presentation, a high GGG, rapid progression after androgen deprivation, a low PSA level relative to the tumor burden, and visceral metastasis [21]. Later, the molecular signature of AVPC has also been defined by the presence of two or more alterations in *PTEN*, *TP53,* and *RB1* (referred to as the AVPC molecular signature, AVPC-ms) [22]. In this regard, we analyzed the frequencies of *TP53, RB1*, or *PTEN* alterations in our cohort. We found that 36 patients (36%) had two or more alterations in these three genes. When their clinical characteristics were compared with the remaining cases, patient age, TNM stage distribution, and PSA at presentation did not show significant differences (Table 6). The ratio of GGG 5 tumors was also not significantly different. We further analyzed this group by separating them into the synchronous metastasis (M1 at presentation, n = 64) and asynchronous disease (M0 at presentation, n = 35) subgroups, but we did not observe any noticeable clinical or pathological differences between *PTEN-*, *TP53-*, or *RB1*-altered AVPCs and non-AVPCs (Supplementary Tables S1 and S4).

### 3.5. Progression of Prostate Cancer and ctDNA NGS Positivity

Next, we compared the disease status between ctDNA-negative and ctDNA-positive mCRPCs. The median PSA for the ctDNA-negative group was 12.0 compared with that of 75.0 for the ctDNA-positive group; however, this difference was not statistically significant (Table 7). Similarly, there was no significant difference in PSA progression status. In contrast, both the radiographic and clinical progression statuses were significantly associated with ctDNA negativity/positivity. Notably, mCRPCs at radiographic progression exhibited a 74.5% likelihood of being ctDNA-positive, whereas mCRPCs at clinical progression demonstrated an 86.2% probability of ctDNA positivity.

## 4. Discussion

In our real-world single-institution cohort, ctDNA positivity was observed in 69% of the mPC cases. Compared to previous studies that conducted ctDNA NGS research in clinical trials or as a part of the real-world clinical practice, no significant differences in patient age, PSA level, or the distribution of metastases were found. Our cohort had a higher number of systemic therapies administered for mCRPC. This study is significant because it provides actual clinical data.

Our study chose ctDNA NGS for several reasons. First, there is a time difference between the collection of tissue samples and the acquisition of ctDNA. ctDNA samples were obtained at the time of disease progression, allowing for a more accurate representation of the real-time tumor burden in patients. Second, it has been reported in numerous previous studies that formalin fixation and paraffin embedding can cause artifacts by affecting the DNA [23,24,25]. Furthermore, the ctDNA analysis workflow and analysis methods we used were validated in our earlier studies [13,26]. The use of chemotherapy can influence ctDNA positivity, and, therefore, it is intriguing that 51 out of 74 mCRPC patients (69%) tested positive for ctDNA despite a high number of patients who were exposed to docetaxel, cabazitaxel, or PARP inhibitors.

In our efforts to identify and validate the key genetic alterations using a ctDNA NGS analysis, a critical parameter was the depth of the sequencing performed. Our study incorporated a substantial average sequencing depth of over 30,000×. This extensive depth is significant in increasing the confidence in the accuracy of our variant detection. Furthermore, it empowered our study to identify rarer or low-frequency mutations with greater sensitivity.

In the patients with mCRPC, the probability of ctDNA positivity was higher during radiographic or clinical progression. However, there was no significant correlation with PSA elevation nor were there significant differences in the PSA medians between the ctDNA-positive and ctDNA-negative groups. Determining when to obtain a ctDNA sample can be challenging, especially during the mCRPC stage when tumor somatic profiling is crucial, as ctDNA levels fluctuate with disease progression like PSA. Our results support the hypothesis that ctDNA sampling during radiographic or clinical progression maximizes the chances of identifying significant mutations. The lack of a significant correlation with PSA progression can be explained as follows: Unlike localized prostate cancer, where PSA shows notable sensitivity/specificity, PSA dynamics often fail to reflect the disease progression status at the mCRPC stage [27]. In our study, 19 patients had PSA levels of <4.0 ng/mL, and among them, 4 had PSA progression, 10 had radiographic progression, and 7 had clinical progression. In the real world, diagnosing PSA progression in patients with low PSA levels can be ambiguous, and clinicians tend to defer the diagnosis until the PSA reaches a certain level before considering treatment changes. In addition, approximately 10–20% of mCRPCs appear as neuroendocrine prostate cancer (NEPC) or double-negative prostate cancer (DNPC), which do not reliably express PSA [28,29,30]. The presence of these subtypes explains why PSA dynamics may not accurately reflect disease progression. Given the mutation frequencies of *TP53* and *RB1* in our study, we anticipated a considerable proportion of NEPCs and DNPCs and poor responses to ARSIs. We believe that ctDNA could be used for determining treatment methods and monitoring treatment responses when serially monitored in patients.

Germline or somatic profiling is insufficient to identify all clinically significant *BRCA1/2* alterations. Since *BRCA1/2* mutations imply a poor prognosis for ARSIs and a favorable response to PARP inhibitors in mCRPC, it is essential to perform both somatic and germline profiling in patients with mPC [31,32]. Previous clinical trials incorporating PARP inhibitors reported the prevalence of *BRCA1/2* mutations in their patient cohorts to be 3.8–7.7% [3,33,34,35]. In comparison to these previous studies, our study reported a relatively high frequency of *BRCA1/2* mutations. *BRCA2* mutations can manifest as SNVs, small indels, and large structural variations or CNVs. Our unique methodology for analyzing ctDNA allowed us to accurately report CNVs, including shallow deletions. These findings align with the results of previous tissue NGS studies on *BRCA2* mutations [27,36].

In this report, we reaffirmed our previous findings that synchronous metastatic prostate cancers with *BRCA1/2* mutations present with relatively low serum PSA levels but higher Gleason grade groups [30]. We previously analyzed our tissue NGS datasets and publicly available mPC datasets, concluding that *BRCA1/2*-mutated metastatic tumors differ from wild-type tumors in PSA levels at diagnosis. It is recommended to perform NGS-based *BRCA1/2* somatic and germline testing when evaluating advanced prostate cancer patients to identify potential familial cancers and to utilize PARP inhibitors or platinum-based chemotherapies when the disease progresses to mCRPC [2,37]. Our findings strongly advocate for somatic and germline testing including *BRCA1/2* in the evaluation of prostate cancer, especially in cases of metastasis at presentation and a Gleason grade group 5 disease, even if their PSA is lower than 100 ng/mL.

Our patient cohort included individuals exposed to chemotherapy. Indeed, chemotherapy can influence ctDNA presence in the blood, potentially affecting the results. Our intention in this study was to depict real-world clinical scenarios where patients with metastatic prostate cancer often receive various combinations of therapies, including chemotherapy. The variability in treatments and the discrepancies in how each patient responds are practical challenges clinicians face. We believe our study mirrors these real-world conditions, making our findings relevant and applicable.

Our study has several limitations. First, the relatively small sample size limits the statistical power of our findings, which restricts the subgroup analysis and the generalizability of our results to a broader population. In addition, the study was conducted in a single institution, potentially reflecting the specific practices or population characteristics of our institution. The findings may not be applicable to other settings; hence, future multicenter studies would be beneficial in this regard. Another limitation is the lack of diversity in our study population, which purely consists of Asian people. The findings may not be directly applicable to populations with different demographic or health profiles. We also did not routinely perform tissue NGS, which may have resulted in false-negative results. Th specific genes that correlate with disease progression remain unidentified. In addition, we were unable to perform a subgroup analysis based on prior drug exposure due to the small sample size. Nevertheless, ctDNA NGS successfully detected significant somatic variants with a probability of approximately 70%. Further studies are needed to examine ctDNA dynamics in response to cancer treatment as well as to identify the genes responsible for disease progression and an increased metastatic burden.

## 5. Conclusions

Our study provides valuable insights regarding the use of ctDNA NGS in the real-world clinical practice for managing mCRPC. These findings emphasize the potential of ctDNA positivity as a marker of disease progression, particularly in the context of radiographic and clinical progression independent of PSA dynamics. Furthermore, we highlight the importance of comprehensive germline and somatic profiling in capturing critical *BRCA1/2* alterations. Despite some limitations, such as potential false negatives, our study reinforces the high detection rate of significant somatic variants and demonstrates the potential of ctDNA NGS in guiding personalized treatment strategies and monitoring therapy responses, particularly in challenging mCRPC cases. Future studies should further validate our findings and explore the broader applicability of ctDNA NGS in the management of advanced prostate cancer.

## Figures and Tables

**Table 1:** Patient characteristics.

Parameter	mHSPC (n = 23)	mCRPC (n = 77)	Total (n = 100)
**Age at time of ctDNA collection, median, years (range)**	69 (57–87)	72 (51–90)	71.5 (51–90)
**PSA at time of ctDNA collection, median, ng/mL (range)**	80.90 (0.3–3110)	52.1 (<0.01 to >5000)	62.9 (<0.01 to >5000)
**Synchronous metastases, n (%)**	23 (100.0%)	40 (51.9%)	63 (63.0%)
**Gleason grade group, n (%)**			
1 to 3	3 (13.0%)	4 (5.2%)	7 (7.07%)
4	10 (43.4%)	19 (24.7%)	29 (29.3%)
5	10 (43.4%)	53 (68.9%)	63 (63.6%)
unidentified	0 (0.0%)	1 (1.3%)	1 (1.01%)
**Metastatic sites at time of ctDNA collection, n (%)**			
bone	22 (95.7%)	76 (98.7%)	97 (97.0%)
lymph node	13 (56.5%)	38 (49.4%)	51 (51.0%)
liver	0 (0.0%)	11 (14.3%)	11 (11.0%)
lung	3 (13.0%)	7 (9.1%)	10 (10.0%)
other	0 (0.0%)	9 (11.7%)	9 (9.0%)
**Prior drug exposure, n (%)**			
abiraterone	5 (21.7%)	42 (54.5%)	47 (47.0%)
enzalutamide	0 (0.0%)	15 (19.5%)	15 (15.0%)
docetaxel	1 (4.8%)	32 (42.6%)	33 (33.0%)
cabazitaxel	0 (0.0%)	3 (3.9%)	3 (3.0%)
PARP inhibitor	0 (0.0%)	2 (2.6%)	2 (2.0%)
**Completed systemic therapy, n (%)**			
1 or less	0 (0.0%)	54 (70.1%)	
2	0 (0.0%)	17 (22.1%)	
3 or more	0 (0.0%)	6 (7.8%)	

ctDNA represents circulating tumor DNA, mHSPC represents metastatic hormone-sensitive prostate cancers, mCRPC represents metastatic castration-resistant prostate cancer, and PSA represents prostate-specific antigen.

**Table 2:** *BRCA1* and *BRCA2* Genetic Alterations in Metastatic Prostate Cancer.

	Germline Variants	Somatic Variants	Total Variants
mCRPC (n = 77)	7 (9.1%)	11 (14.3%)	18 (23.4%)
mHSPC (n = 23)	1 (4.3%)	4 (19.0%)	5 (21.7%)
mPC (n = 100)	8 (8.0%)	15 (15.0%)	23 (22.0%)

mHSPC represents metastatic hormone-sensitive prostate cancers, mCRPC represents metastatic castration-resistant prostate cancer, and mPC represents metastatic prostate cancer.

**Table 3:** Clinical and pathologic characteristics of *BRCA1/2*-altered * metastatic prostate cancers.

	*BRCA1/2* Mutated N = 23	*BRCA1/2* Wild Type N = 76	*p* Value
Age at diagnosis (years) mean ± SD	69.4 ± 6.71	67.2 ± 8.28	0.244 ^1^
PSA at diagnosis (ng/mL) median (range)	55.8 (3.9–1422)	117.1 (3.56–5000)	0.123 ^2^
Gleason grade group, N (%) ^†^			0.094 ^3^
1–4	7 (30.4%)	40 (52.6%)	
5	16 (69.6%)	36 (47.4%)	

* *BRCA1/2* alterations include both germline and somatic alterations; ^†^ one patient did not have their Gleason grade group nor their TNM stage reported. ^1^ Student’s *t*-test; ^2^ Mann–Whitney test; ^3^ chi-square test.

**Table 4:** Clinical and pathologic characteristics of *BRCA1/2*-altered * synchronous metastatic prostate cancers.

	*BRCA1/2* Mutated N = 12	*BRCA1/2* Wild Type N = 52	*p* Value
Age at diagnosis (years) mean ± SD	70.6 ± 7.44	67.5 ± 7.67	0.206 ^1^
PSA at diagnosis (ng/mL) median (range)	61.8 (4.50–1422)	224.4 (5.33–5000)	0.034 ^2^
Gleason grade group, N (%) ^†^			0.022 ^3^
1–4	2 (16.7%)	29 (56.9%)	
5	10 (83.3%)	22 (43.1%)	

* *BRCA1/2* alterations include both germline and somatic alterations; ^†^ one patient did not have their Gleason grade group nor their TNM stage reported. ^1^ Student’s *t*-test; ^2^ Mann–Whitney test; ^3^ chi-square test.

**Table 5:** Clinical and pathologic characteristics of *BRCA1/2*-altered * asynchronous metastatic prostate cancers.

	*BRCA1/2* Mutated N = 11	*BRCA1/2* Wild Type N = 24	*p* Value
Age at diagnosis (years) mean ± SD	68.2 ± 5.91	67.4 ± 9.11	0.801 ^1^
PSA at diagnosis (ng/mL) median (range)	50.9 (3.9–390.3)	20.0 (3.56–333.3)	0.224 ^2^
Gleason grade group, N (%) ^†^			0.833 ^3^
1–4	5 (45.5%)	10 (41.7%)	
5	6 (54.5%)	14 (58.3%)	

* *BRCA1/2* alterations include both germline and somatic alterations; ^†^ one patient did not have their Gleason grade group nor their TNM stage reported. ^1^ Student’s *t*-test; ^2^ Mann–Whitney test; ^3^ chi-square test.

**Table 6:** Clinical and pathologic characteristics of metastatic aggressive-variant prostate cancer (AVPC).

	AVPC N = 36	Non-AVPC N = 63	*p* Value
Age at diagnosis (years) mean ± SD	67.8 ± 7.91	68.0 ± 7.80	0.900 ^1^
PSA at diagnosis (ng/mL) median (range)	33.0 (3.9–5000)	121.1 (3.56–3110)	0.089 ^2^
Gleason grade group, N (%) ^†^			0.196 ^3^
1–4	14 (38.9%)	33 (52.4%)	
5	22 (61.1%)	30 (47.6%)	

^†^ one patient did not have their Gleason grade group nor their TNM stage reported. ^1^ Student’s *t*-test; ^2^ Mann–Whitney test; ^3^ chi-square test.

**Table 7:** Prostate cancer disease progression status and ctDNA positivity.

mCRPC pts (n = 74)		ctDNA Negative (n = 23)	ctDNA Positive (n = 51)	*p* Value
PSA (ng/mL) Median (range)		12.0 (0.1–5000)	75.0 (0.1–5000)	0.0844 *
PSA progression (row %)	No	11 (40.7)	16 (59.3)	0.1996 ^†^
	Yes	12 (25.5)	35 (74.5)	
Radiographic progression (row %)	No	9 (69.2)	4 (30.8)	0.0022 ^†^
	Yes	14 (22.9)	47 (77.1)	
Clinical progression (row %)	No	19 (42.2)	26 (57.8)	0.0111 ^†^
	Yes	4 (13.8)	25 (86.2)	

* Mann–Whitney U test; ^†^ Fisher’s exact test. mCRPC represents metastatic castration-resistant prostate cancer; ctDNA represents circulating tumor DNA.

## Data Availability

Hyunho Han had full access to all the data in this study and takes responsibility for the integrity of the data and the accuracy of the data analysis.

## Supplementary Materials

The following supporting information can be downloaded at https://www.mdpi.com/article/10.3390/cancers15153998/s1, Table S1: Patient demographics; Table S2: Somatic gene alterations; Table S3: Gene panel information. Table S4: BRCA, AVPC.
